# CHARACTERISTICS OF HOSPITALIZED OLDER ADULTS WITH RECURRENT CLOSTRIDIUM DIFFICILE: INTERVENTION OPPORTUNITIES

**DOI:** 10.1093/geroni/igz038.1677

**Published:** 2019-11-08

**Authors:** Anna Boone, Laurie Kennedy-Malone, Thomas McCoy, Deborah A Lekan, Debra Wallace, Robert Rourk

**Affiliations:** 1 Cone Health, Reidsville, North Carolina, United States; 2 University of North Carolina at Greensboro, Greensboro, North Carolina, United States; 3 UNC-Greensboro School of Nursing, Greensborol, North Carolina, United States

## Abstract

Clostridium difficile infection (CDI) is the leading cause for gastroenteritis-associated deaths (Hall et al., 2012). Risk factors include advanced age, antibiotic use, and hospital admission, yet multiple others are not widely known. CDI recurrence risk may be as high as 40% after initial treatment (Garey et al., 2008; Kelly & Lamont, 2008). Older adults may present atypically, and treatment guidelines for initial and recurrent CDI have evolved from older standards. A retrospective cohort research study explored characteristics of hospitalized adults ages 55 and older with CDI between December 31, 2013 through December 31, 2015, identified by ICD diagnosis codes. Recurrence within one year, laboratory measurements, chronic diseases, and psychosocial data were captured from the electronic health record (EHR). Over the study period, 871 patients had a recurrence rate of 23.9% (n=208). Caucasian females comprised over half the sample, and 9.1% expired during initial hospitalization. Almost two-thirds (n=576, 66.1%) lived in private residences prior to admission. CDI recurrence was more prevalent if discharged to skilled nursing facility and home health care services. Hypertension, heart failure, and chronic kidney disease were most prevalent in the recurrent CDI group. Polypharmacy was noted in over two-thirds of sample. A large portion of the sample displayed hypoalbuminemia on admission.Utilizing the EHR to aggregate data promotes interventions to reduce recurrence, prolonged stay, and aggressive treatment per current guidelines. Multidisciplinary approaches include deprescribing, nutritional support, and chronic disease management. Person-centered care and individualized interventions should begin on admission with close outpatient follow-up and hopeful reduced readmissions.

